# cAMP-mediated autophagy inhibits DNA damage-induced death of leukemia cells independent of p53

**DOI:** 10.18632/oncotarget.25758

**Published:** 2018-07-13

**Authors:** Seham Skah, Nina Richartz, Eva Duthil, Karin M. Gilljam, Christian Bindesbøll, Elin Hallan Naderi, Agnete B. Eriksen, Ellen Ruud, Marta M. Dirdal, Anne Simonsen, Heidi Kiil Blomhoff

**Affiliations:** ^1^ Department of Molecular Medicine, Institute of Basic Medical Sciences, University of Oslo, Oslo, Norway; ^2^ Department of Oncology, Section for Head and Neck Oncology, Oslo University Hospital, Oslo, Norway; ^3^ Department of Hematology and Oncology, Division of Pediatric and Adolescent Medicine, Oslo University Hospital, Oslo, Norway; ^4^ Institute of Clinical Medicine, Faculty of Medicine, University of Oslo, Oslo, Norway; ^5^ Centre for Cancer Cell Reprogramming, Institute of Clinical Medicine, Faculty of Medicine, University of Oslo, Oslo, Norway

**Keywords:** cAMP-signaling, autophagy, DNA damage, p53, apoptosis

## Abstract

Autophagy is important in regulating the balance between cell death and survival, with the tumor suppressor p53 as one of the key components in this interplay. We have previously utilized an *in vitro* model of the most common form of childhood cancer, B cell precursor acute lymphoblastic leukemia (BCP-ALL), to show that activation of the cAMP signaling pathway inhibits p53-mediated apoptosis in response to DNA damage in both cell lines and primary leukemic cells. The present study reveals that cAMP-mediated survival of BCP-ALL cells exposed to DNA damaging agents, involves a critical and p53-independent enhancement of autophagy. Although autophagy generally is regarded as a survival mechanism, DNA damage-induced apoptosis has been linked both to enhanced and reduced levels of autophagy. Here we show that exposure of BCP-ALL cells to irradiation or cytotoxic drugs triggers autophagy and cell death in a p53-dependent manner. Stimulation of the cAMP signaling pathway further augments autophagy and inhibits the DNA damage-induced cell death concomitant with reduced nuclear levels of p53. Knocking-down the levels of p53 reduced the irradiation-induced autophagy and cell death, but had no effect on the cAMP-mediated autophagy. Moreover, prevention of autophagy by bafilomycin A1 or by the ULK-inhibitor MRT68921, diminished the protecting effect of cAMP signaling on DNA damage-induced cell death. Having previously proposed a role of the cAMP signaling pathway in development and treatment of BCP-ALLs, we here suggest that inhibitors of autophagy may improve current DNA damage-based therapy of BCP-ALL - independent of p53.

## INTRODUCTION

Improved awareness of the vital cellular process of autophagy has in recent years enhanced our understanding of cancer development as well as mechanisms underlying resistance to cancer treatment [1–4]. Macroautophagy, hereafter referred to as autophagy, involves bulk degradation of cytoplasmic components like damaged organelles and long-lived proteins. A double-membraned vesicle, the autophagosome, forms as it sequesters cargo destined for degradation, and the content is degraded after fusion between the autophagosomes and lysosomes [5, 6]. Multiple key proteins have been implicated in the various steps of the autophagic process, including Unc-51 like autophagy activating kinase (ULK1) involved in the early steps of autophagophore formation [7], and the microtubule-associated protein1 (MAP1) light chain 3 (LC3) widely used as a marker for assessing autophagic flux [8, 9].

Autophagy is required for cells and tissues to maintain homeostasis at critical times of energy demand and cellular stress, and it is considered to be an important regulator of the balance between cell death and cell survival [10–12]. Although autophagy is generally regarded as a survival mechanism, extensive autophagy has also been linked to cell death. Numerous studies have shown that autophagy may either promote or prevent cell death in response to DNA damage [10, 13, 14]. Most studies however, conclude that inhibition of autophagy results in enhanced DNA damage-induced apoptosis, supporting a protective role for autophagy in the DNA damage response (DDR) [13].

In the present study, we reveal the interplay between DDR, p53, apoptosis, and autophagy in leukemia cells. B-cell precursor acute lymphoblastic leukemia (BCP-ALL) is the most common form of pediatric cancers [15]. Despite the general favorable survival rate of children with BCP-ALL, there is ongoing research to improve the treatment efficiency of subgroups with poor prognosis [15]. The poor prognostic group of BCR/ABL1 positive BCP-ALLs appears particularly dependent on autophagy for their survival and malignant transformation [16]. Treatment of lymphoid malignancies with DNA damaging anti-cancer agents will induce cell cycle arrest, DNA repair, apoptosis or autophagy depending on the balance between these processes. A higher level of autophagy is generally associated with worse clinical outcome [17]. In line with this notion, it has been reported that inhibition of autophagy overcomes treatment resistance in lymphoid malignant cells [18].

There are multiple mechanisms proposed to explain how DNA damage promotes autophagy, including activation of ataxia-telangiectasia mutated (ATM) [19] and induction of nuclear p53 [20, 21]. BCP-ALLs develop in the bone marrow in close contact with stromal cells that produce prostaglandin E2 (PGE2) [22], and BCP-ALL cells also express functional PGE2 receptors (EP2) [23]. We have previously shown that PGE2 produced by residential stromal cells in the bone marrow limits DNA damage-induced p53 levels via activation of the cAMP signaling pathway, and we have proposed that this may have detrimental effects on both development and treatment of BCP-ALL [24]. Thus, we have shown that cAMP signaling inhibits p53-mediated apoptosis of BCP-ALL cells exposed to irradiation or cytotoxic drugs [24–26]. Here, we have uncovered a novel p53-independent link between cAMP-mediated enhancement of autophagy and its ability to reduce DNA damage-induced apoptosis in BCP-ALL cells.

## RESULTS

### cAMP signaling enhances autophagy induced by DNA damaging agents in REH cells

**Figure 1:**
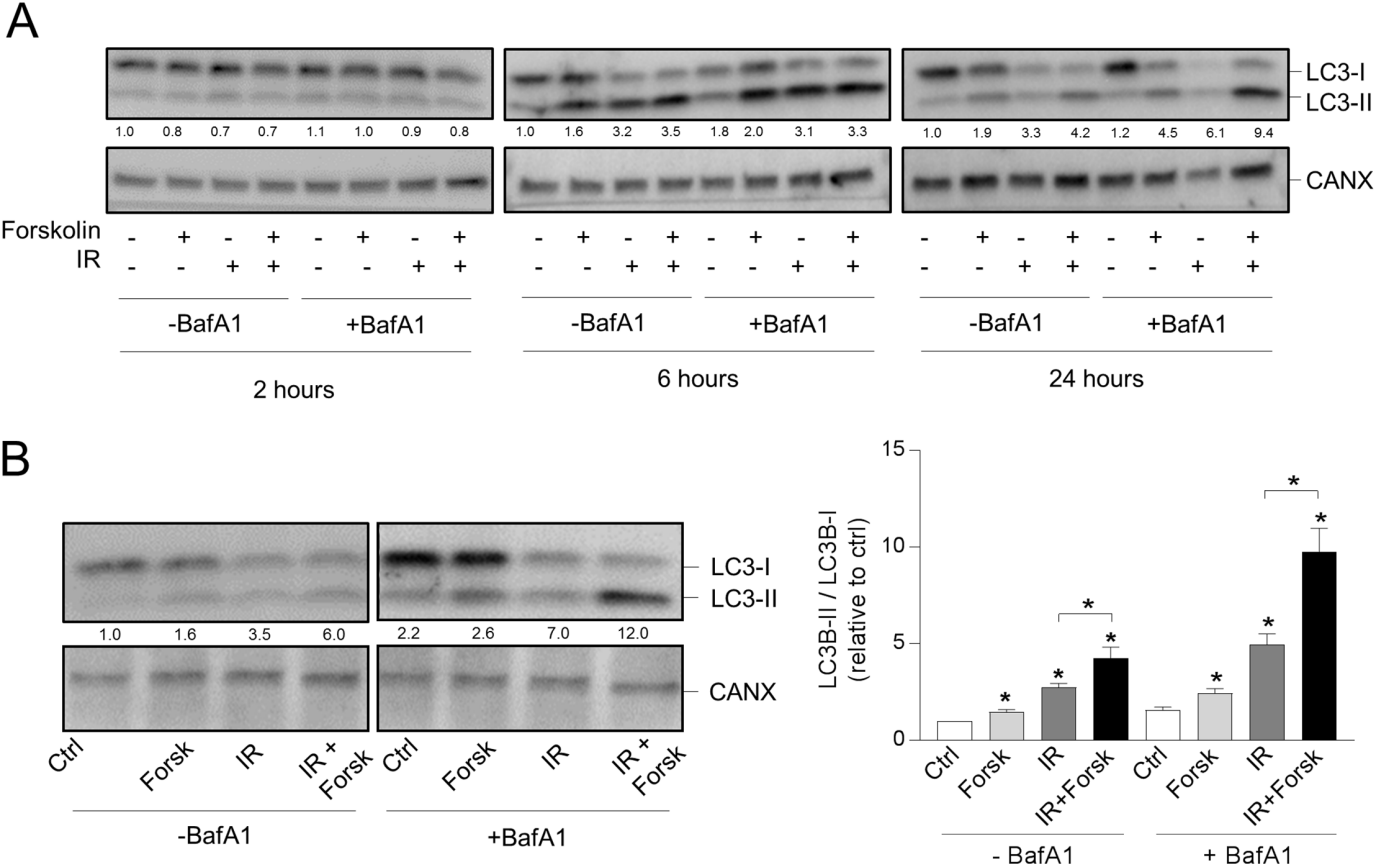
cAMP signaling enhances the DNA damage-induced LC3-II/LC3-I ratio **(A** and **B)** REH cells (0.6×10^6^ cells/ml) were incubated in the presence or absence of forskolin (Forsk, 60 μM) for 45 min prior to irradiation (IR, 10Gy), and total lysates were subjected to immunoblot analyses with antibodies against LC3B or calnexin (CANX). The numbers indicated below the LC3 images represent the LC3-II/LC3-I signal ratios relative to the CANX signals, normalized to the ratio in untreated (Ctrl) cells. (A) When indicated, BafA1 (2 nM) was added from the start of the cultures. The cells were harvested at the indicated time points after IR, and one representative Western blot of three is shown. (B) Left panel: The cells were harvested 24 hours after IR, and BafA1 (2 nM) was added for the last 4 hours, as indicated. One representative Western blot of 8 is shown. Right panel: Ratios of the LC3-II/LC3-I signal intensities relative to the CANX signals, normalized to ratio in untreated (Ctrl) cells. The data represent the mean +/- SEM, n=8. ^*^p< 0.05 (paired *t* test).

We have previously shown that activation of the cAMP signaling pathway limits DNA damage-induced apoptosis in BCP-ALL cell lines as well as in primary leukemic cells [24, 26, 27]. Here we aimed to elucidate whether cAMP-mediated survival of BCP-ALL cells involves enhanced autophagy. To test this hypothesis, we treated the ALL cell line REH with the adenylate cyclase activator forskolin, at the optimal concentration of 60 μM, followed by X-ray-mediated irradiation (IR) at 10 Gy. To measure autophagic flux we took advantage of the well-established marker of phagophores and autophagosomes, LC3B. Upon induction of autophagy, the cytosolic form of LC3B (LC3-I) becomes conjugated to phosphatidylehtanolamine (PE) in phagophore membrane and converted to LC3-II. Because the two forms run at different molecular weights when analyzed by western blotting, the LC3-II/I ratio normalized to loading control is therefore commonly used to assess the formation of autophagosomes [8, 9]. As shown in Figure 1A, both IR and forskolin alone induced autophagosome accumulation as assessed by the enhanced LC3-II/LC3-I ratio. The effect of forskolin on LC3-II formation was stronger than that of IR alone and was notable after 6 hours, but more pronounced after 24 hours. Forskolin markedly enhanced the IR-induced LC3-II/I ratio, most prominent after 24 hours.

Accumulation of autophagosomes can be the result of either induced formation of autophagosomes (induced autophagic flux) or be due to blocked autophagosome degradation [8]. To distinguish between these two possibilities, the same experiments were performed in the presence of the lysosomal inhibitor bafilomycin A1 (BafA1). BCP-ALL cells are known to be sensitive to BafA1-treatment [28], and dose response experiments revealed that 2 nM of BafA1 was the optimal non-toxic concentration for REH cells (data not shown). As shown in Figure 1A, the LC3-II/I ratios induced by IR and/or forskolin were clearly enhanced by BafA1 - suggesting enhanced autophagic flux. In Figure 1A, BafA1 was added from the start of the culture. However, to avoid adverse effects of the inhibitor, we also assessed the LC3-II/I ratios after shorter exposure to BafA1. As shown in the left panel of Figure 1B, we concluded that it was sufficient with 2 nM of BafA1 for the last 4 hours prior to cell harvesting. When using these conditions, we found that forskolin significantly (p<0.01) enhanced the IR-induced LC3-II/I ratio from 4.95 to 9.78 (Figure 1B, right panel). Taken together, we have shown that forskolin and IR independently induces autophagy, and that forskolin is able to potentiate the irradiation-induced autophagy.

### Protein kinase a mediates the effects of forskolin

**Figure 2:**
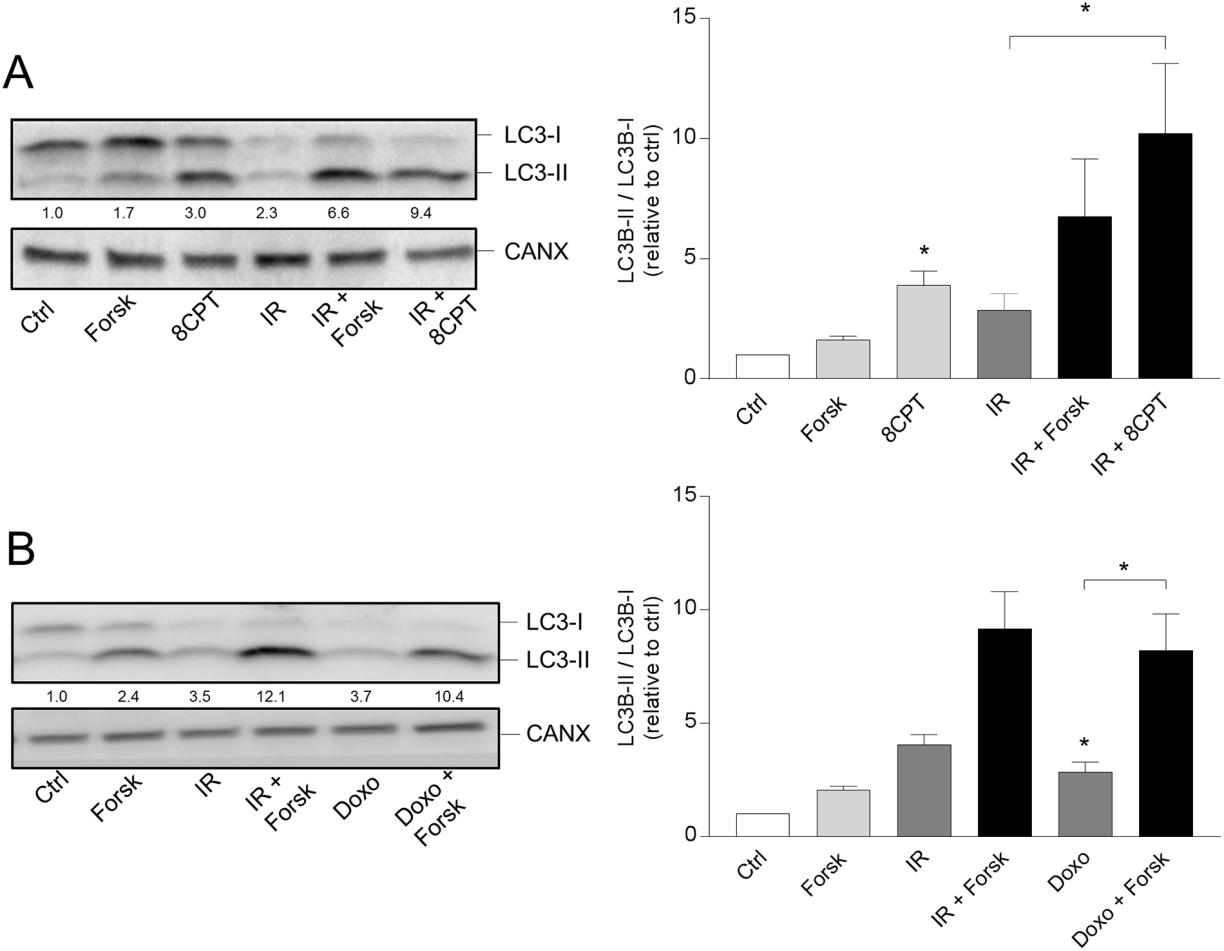
PKA- and doxorubicin-mediated autophagy **(A** and **B)** REH cells were treated with or without forskolin, IR and BafA1 as described in Figure 1B. When indicated, the cells were treated with or without 8CPT-cAMP (8CPT, 200μM) 45 min prior to IR (panel A) or with 150 nM doxorubicin (Doxo) 45 min after adding forskolin (panel B). Left panels: One representative Western blot of three independent experiments is shown. The numbers indicated below the LC3 images represent the LC3-II/LC3-I signal ratios relative to the CANX signals, normalized to the ratio in untreated (Ctrl) cells. Right panels: Ratios of the LC3-II/LC3-I signal intensities relative to the CANX signals, normalized to the ratio in untreated (Ctrl) cells. The data represent the mean +/- SEM, *n*=3. ^*^p<0.05 (paired *t* test).

cAMP signaling induced by forskolin may result in activation of different effector molecules, including protein kinase A (PKA), Epac and cyclin nucleotide-gated cation channels [29]. We previously concluded that forskolin-mediated inhibition of DNA damage-induced apoptosis in BCP-ALL cells is mediated *via* PKA [25]. Here we show that the PKA activator 8-CPT-cAMP induced formation of autophagosomes in the same manner as forskolin – both alone and in the presence of IR (Figure 2A). Furthermore, we showed that the PKA inhibitor RP-8-Br-cAMP reduced the forskolin-mediated enhancement of IR-induced autophagy (Supplementary Figure 1A), and that the phosphodiesterase inhibitor IBMX enhanced the effects of low concentrations of forskolin on autophagy (Supplementary Figure 1B). Autophagy was here quantified by staining the cells with a newly developed dye CYTO-ID, reported to selectively stain autophagocytic vesicles [30]. We also demonstrated that the potentiating effects of cAMP signaling on DNA damage-induced autophagosome formation in REH cells was not limited to IR, but that forskolin also enhanced the LC3-II/I ratio induced by other DNA damaging agents, such as the leukemia relevant drug doxorubicin (Figure 2B).

### cAMP signaling increased the autophagic flux in REH cells

**Figure 3:**
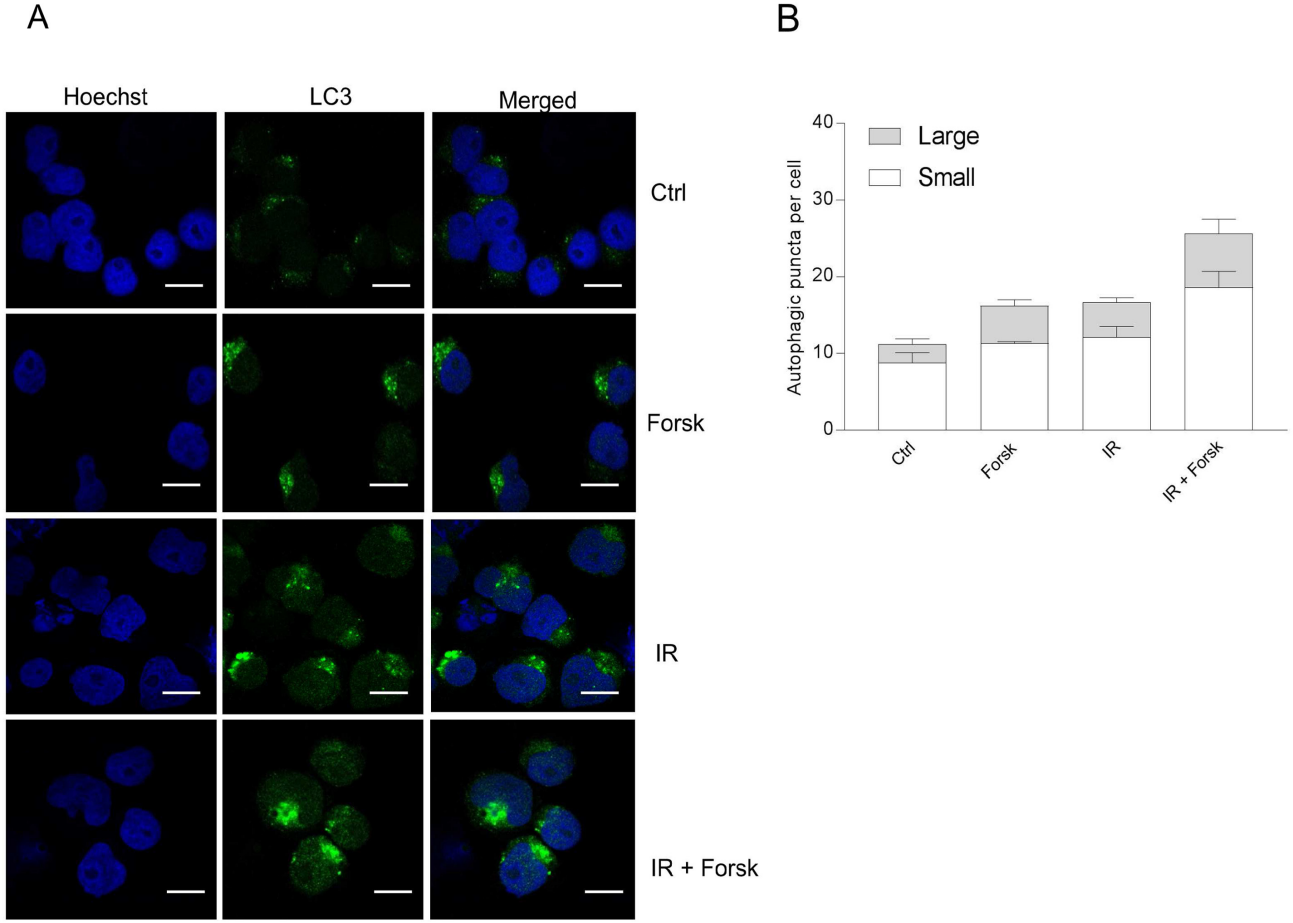
Immunocytochemistry of LC3-puncta **(A** and **B)** REH cells were treated with or without forskolin, IR and BafA1 as described in Figure 1B, with addition of BafA1 to all samples. The cells were subjected to immunocytochemistry for the detection of LC3 puncta by confocal microscopy, and the cells were co-stained with Hoechst for visualization of the nuclei. (A) One representative of three independent experiments is shown. Scale bars = 10μm. (B) The number of LC3 puncta per cell from three independent experiments were quantified, counting at least 30 cells. The data represent the mean +/- SEM, *n* = 30. The numbers of small and large puncta are indicated.

**Figure 4:**
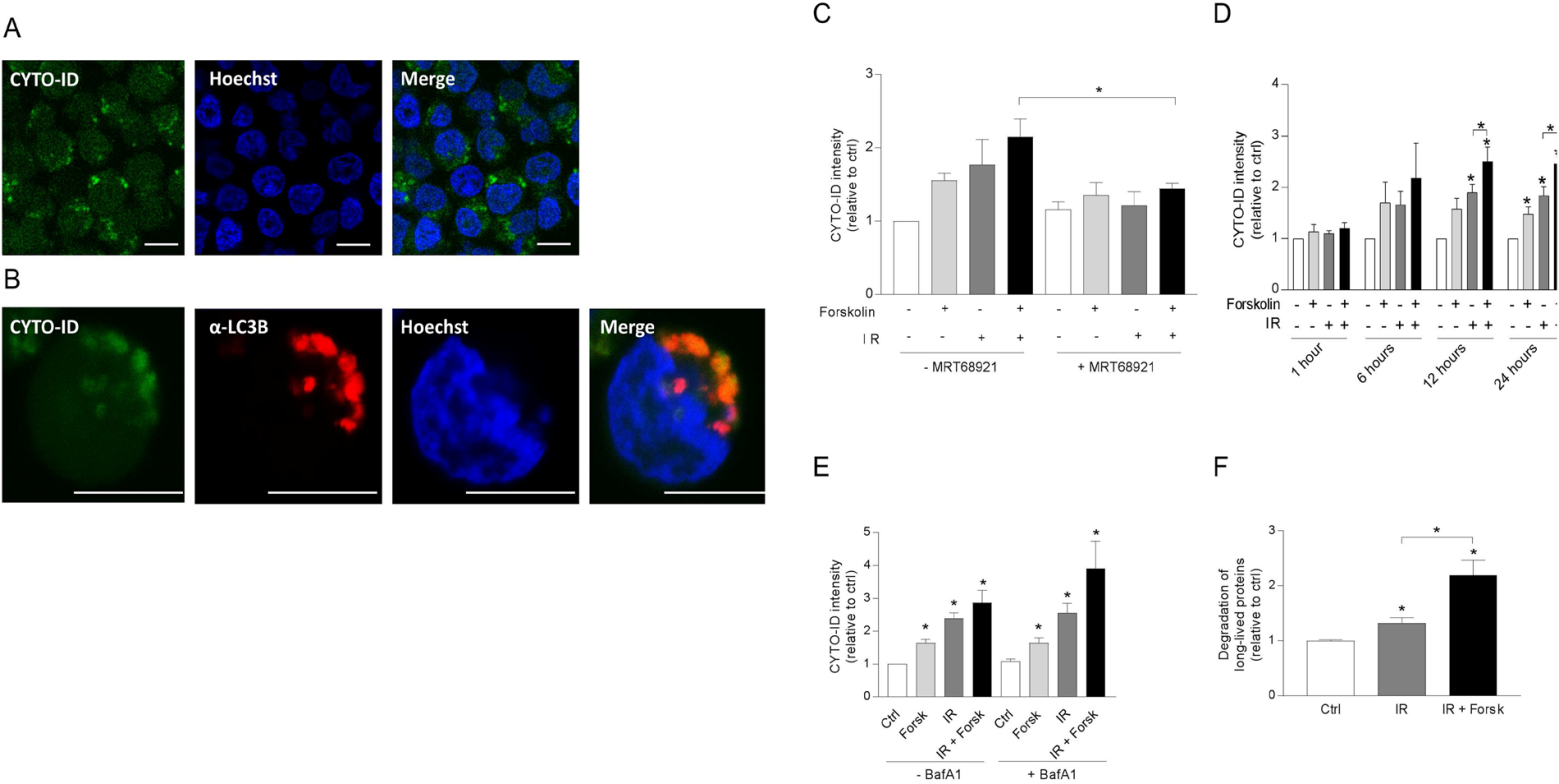
cAMP signaling enhances autophagic flux in REH cells **(A-E)** REH cells were treated with or without forskolin, IR and BafA1 as described in Figure 1B. CYTO-ID staining was performed 24 hours after IR – if not otherwise indicated, and the staining intensity was analyzed by flow cytometry and normalized to untreated (Ctrl) cells. (A) Confocal images of IR/forskolin-treated cells stained with CYTO-ID, (B) The co-localization between the autophagosomal marker CYTO-ID and LC3-puncta was analyzed by confocal imaging of IR/forskolin-treated cells. (C) The cells were pretreated with the ULK1 inhibitor MRT68921 (100 nM) for 30 min prior to adding forskolin, and the cells were irradiated after another 45 min. The data represent the mean CYTO-ID fluorescence intensity +/- SEM, *n*=3. ^*^p=0.05 (paired *t* test). (D) The cells were stained with CYTO-ID at the indicated time points, and the fluorescence intensity was analyzed by flow cytometry. The data represent the mean CYTO-ID fluorescence intensity +/- SEM, *n*=5. ^*^p<0.05 (paired *t* test). (E) Cells were treated with or without 2 nM BafA1for the last 4 hours of the 24 hours incubation. The data represent the mean CYTO-ID fluorescence intensity +/- SEM, *n*=5. ^*^p<0.05 (paired *t* test). **(F)** The effect of IR and forskolin on relative autophagic flux was quantified by measuring the degradation of long-lived proteins as described in Materials and Methods. The data represent the mean +/- SEM, *n*=3, and the values are normalized to the degradation in untreated (Ctrl) cells. ^*^p<0.05 (paired *t* test).

Having demonstrated that cAMP signaling enhances LC3-II formation both alone and in the presence of DNA damaging agents, we next confirmed the formation of autophagosomes by assessing LC3-II puncta by confocal microscopy. As shown in Figure 3, forskolin and IR independently increased the number and sizes of LC3-II puncta after 24 hours, with enhanced levels when the two treatments were combined. We further confirmed the induction of autophagy by staining the cells with CYTO-ID. In Figure 4A, we show CYTO-ID staining of REH cells treated with IR in the presence of forskolin, as revealed by confocal microscopy. The co-localization between CYTO-ID staining and LC3-II puncta is demonstrated in Figure 4B. We demonstrated that pre-incubating the cells for 30 min with the ULK1 inhibitor MRT68921 at the optimal concentration of 100 nM prevented the forskolin-induced CYTO-ID staining as assessed by flow cytometry (Figure 4C). The same effect was observed with siRNA against ULK1 (see Supplementary Figure 2). Careful kinetic experiments concluded that optimal CYTO-ID staining was obtained between 12 and 24 hours, with a clear induction by forskolin noted already after 6 hours of treatment (Figure 4D). Treatment with BafA1 augmented the CYTO-ID staining measured after 24 hours (Figure 4E), enhancing the fold induction of IR-induced CYTO-ID fluorescence intensity from approximately 2.5 to 4. Thus, again we concluded that cAMP signaling enhances DNA damage-induced autophagy.

To further support the cAMP-mediated enhancement of IR-induced autophagy, we measured autophagic flux as the degradation of long-lived proteins, known to be mainly degraded by autophagy [9]. Accordingly, IR alone enhanced the degradation of long-lived proteins in REH cells, and forskolin significantly (p=0.01) enhanced this degradation (Figure 4F).

Autophagy-related genes (ATGs) are differentially regulated at transcriptional, post-transcriptional and post-translational levels [31]. Since IR has been shown to induce transcription of the LC3B-coding gene *MAP1LC3B* [32], we performed qRT-PCR of this gene in REH cells. As shown in Supplementary Figure 3, IR and forskolin alone produced only marginally elevated levels of *MAP1LC3B* mRNA. However, clear additive effects on *MAP1LC3B* mRNA levels were obtained when combining the two treatments. The total level of LC3B protein (LC3-I + LC3-II) was not enhanced in REH cells co-treated by IR and forskolin as compared to control (see Figure 1).

### Forskolin enhances DNA damage-induced autophagy in NALM-6 and primary BCP-ALL cells

**Figure 5:**
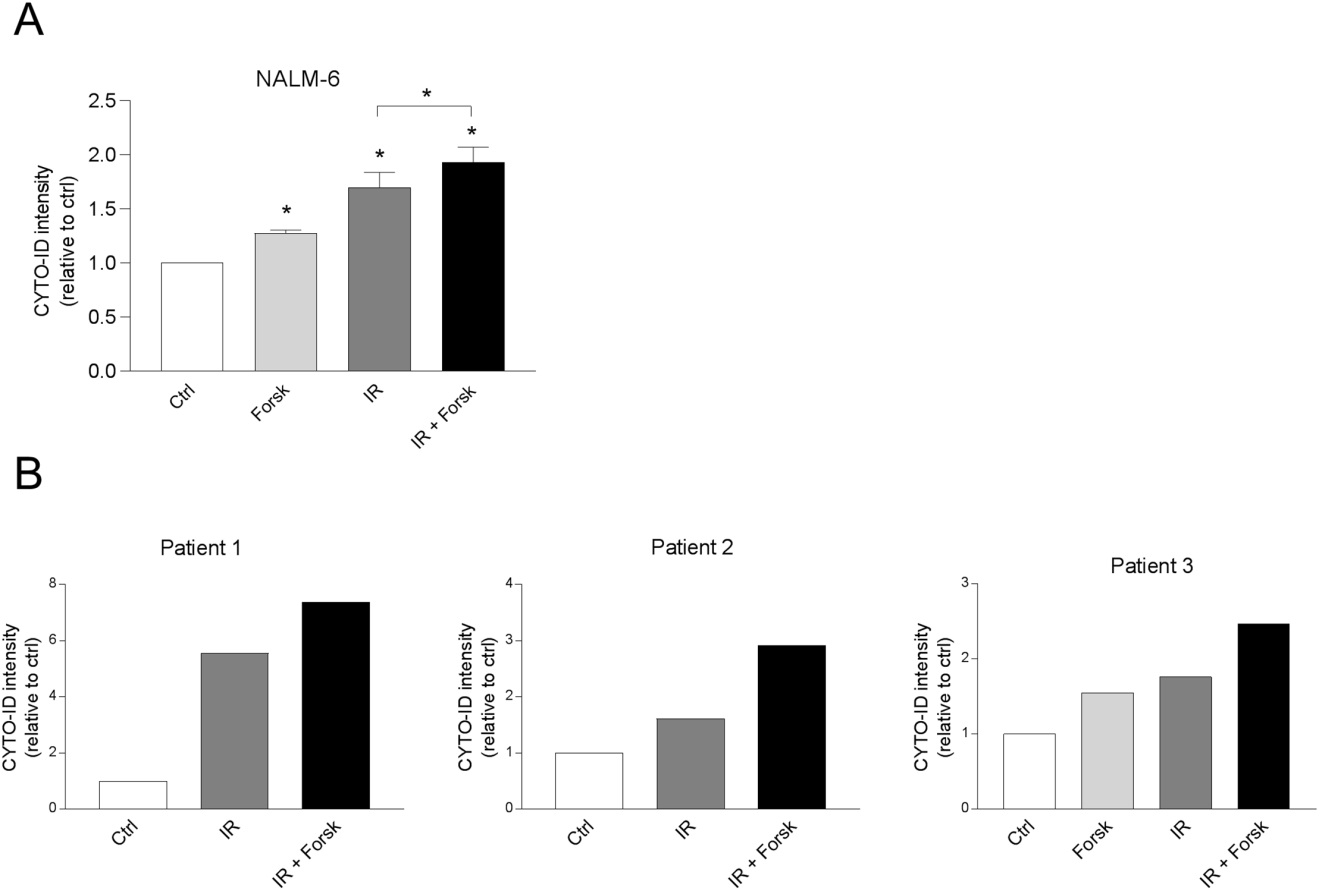
cAMP signaling enhances CYTO-ID staining in NALM-6 and in primary BCP-ALL cells **(A)** NALM-6 cells (0.6×10^6^ cells/ml) were treated with or without forskolin (Forsk, 60μM) for 45 min prior to irradiation (IR, 5Gy). BafA1 (2 nM) was added to the cell cultures for the last 4 hours of the 24 hours incubation, before the cells were stained with CYTO-ID and analyzed for fluorescence intensity by flow cytometry. The data represent the mean CYTO-ID fluorescence intensity ± SEM, *n*=4. ^*^p<0.05 (paired *t* test). **(B)** Primary leukemic blasts (0.6 x10^6^ cells/ml) from three patients diagnosed with BCP-ALL were treated with or without Forsk, IR and BafA1 as described in panel A.

We have previously shown that cAMP signaling regulates DNA damage-induced apoptosis in a similar manner in REH cells, NALM-6 and in primary leukemic cells from patients with BCP-ALL [24]. We therefore investigated whether cAMP signaling also enhanced the IR-induced autophagy in NALM-6, and in primary leukemic cells, using cells from three patients with BCP-ALL. Indeed, the CYTO-ID fluorescence intensity increased in NALM-6 cells when treated with IR in the presence or absence of forskolin (Figure 5A), as was also the case for cells derived from three patients with BCP-ALL (Figure 5B). Due to limited number of cells, we did not assess the ability of forskolin alone to induce autophagy in cells obtained from patient #1 and #2.

### Autophagy is involved in cAMP-mediated survival of DNA damaged cells

**Figure 6:**
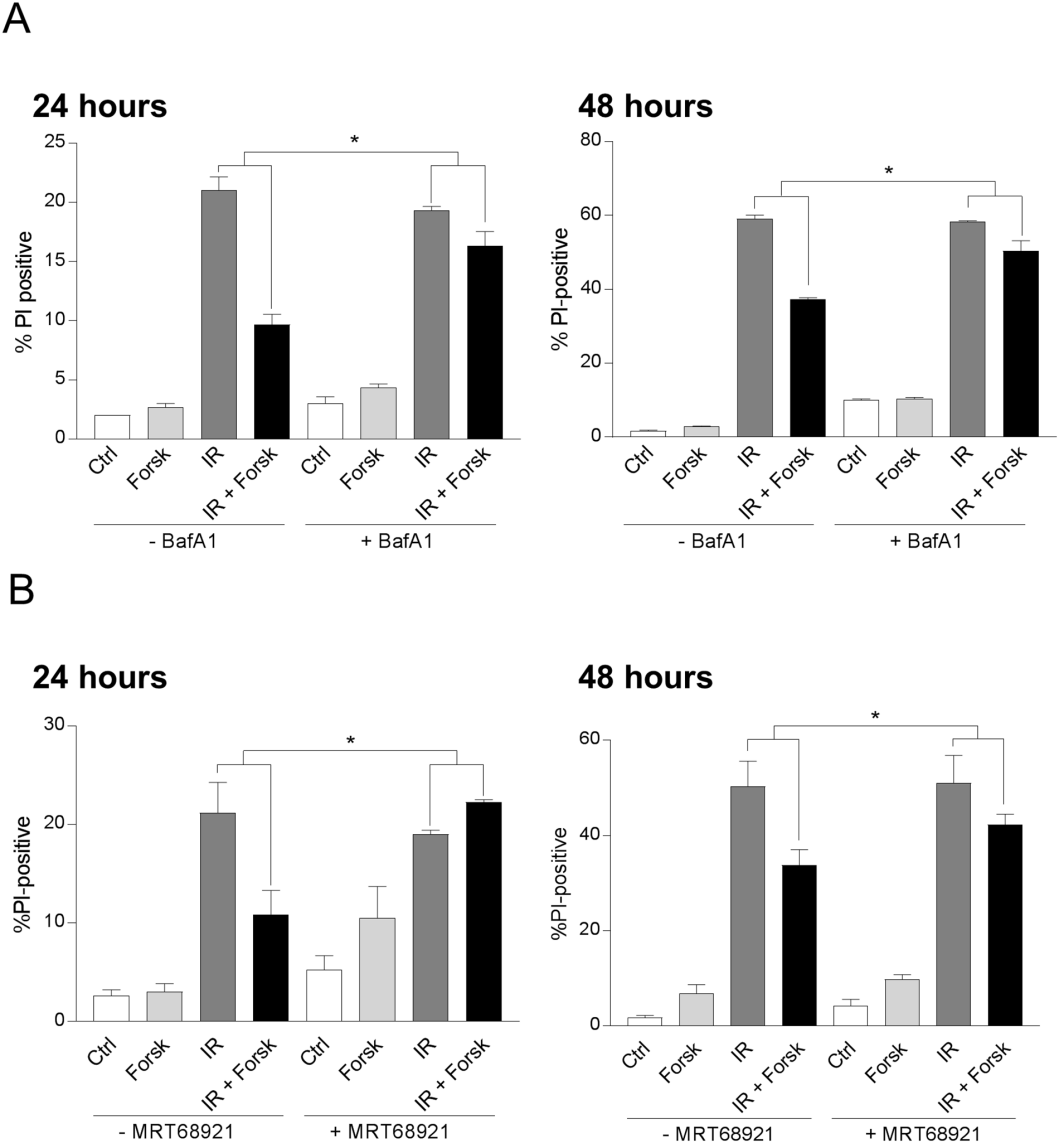
cAMP-mediated inhibition of DNA damage-induced cell death involves autophagy REH cells were treated with or without forskolin and irradiation as described in Figure 1. When indicated, BafA1 (2 nM) **(A)** or the ULK1 inhibitor MRT68921 (100 nM) **(B)** was present in the cell cultures throughout the experiments to block autophagy. The percentage of PI-positive cells was analyzed by flow cytometry 24 hours or 48 hours after IR, as indicated. The results are presented as the mean ± SEM, *n*=6. ^*^p<0.05 (paired *t* test).

Having established the ability of forskolin to enhance the level of DNA damage-induced autophagy, we aimed to identify a possible link between the increased autophagy and the reduced cell death promoted by cAMP signaling in REH cells. As shown in Figure 6A, IR alone induced cell death in approximately 21% of the cells as measured after 24 hours, and in 59% after 48 hours. In line with our previous results [24], forskolin alone had only minor effects on the basal levels of cell death in REH cells, but significantly reduced the IR-induced cell death after 24 hours and 48 hours. To investigate the link between forskolin-induced autophagy and increased survival of the DNA damaged cells, we used inhibitors of autophagy at doses that were not toxic to the cells after 48 hours of treatment, but still retained the ability to prevent autophagic degradation. By using BafA1 at 2 nM from start of the cultures, we found that the forskolin-mediated protection of cell death was significantly reduced after both 24 hours and 48 hours (Figure 6A). The same tendency was observed when treating the cells with 5μM of chloroquine (data not shown). Finally, we demonstrated that inhibiting autophagy by the ULK1 inhibitor MRT68921 impeded the cAMP-mediated protection against DNA damage-induced cell death after both 24 hours and 48 hours (Figure 6B).

### The involvement of p53 in IR-induced autophagy

**Figure 7:**
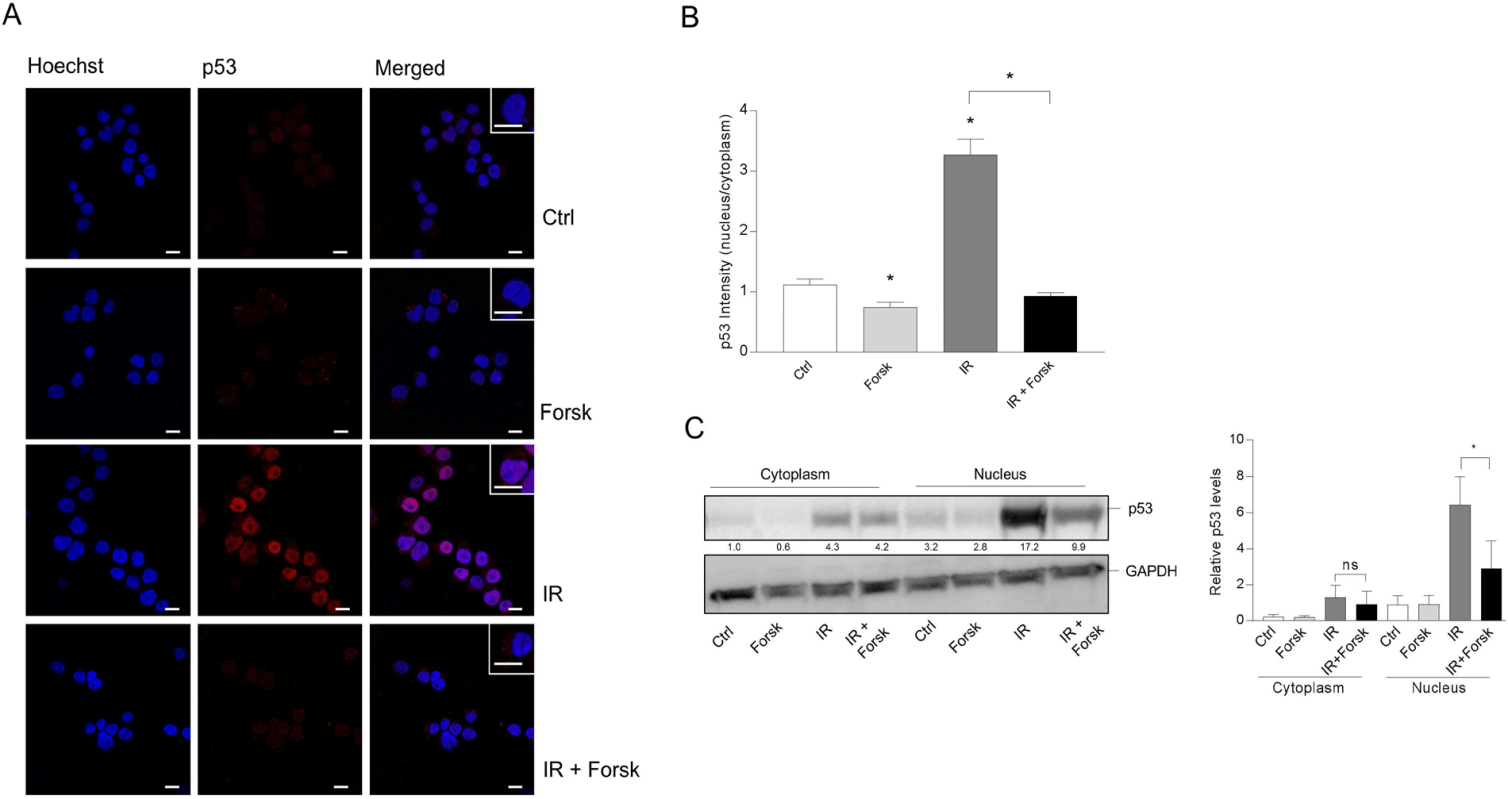
The effects of IR and cAMP signaling on the subcellular localization of p53 REH cells were treated with or without forskolin and IR as described in Figure 1. **(A)** 4 hours after IR, the cells were subjected to immunocytochemistry for the detection of the subcellular localization of p53 by confocal microscopy, and the cells were co-stained with Hoechst for the visualization of nuclei. One representative of three independent experiments is shown. Scale bars = 10μm. **(B)** Quantification of the p53 fluorescence intensity of cells from three experiments, analyzing at least 30 cells. The data represent the mean +/-SEM, *n*=30. ^*^p<0.05, (paired *t* test). **(C)** Subcellular fractionation of REH cells (20×10^6^) was performed as described in Materials and Methods 4 hours after IR. The cytoplasmic and nuclear fractions were each subjected to immunoblot analyses of p53 expression. The numbers indicated below the p53 image represents the p53 signal intensity relative to the CANX signal, normalized to the ratio in untreated (Ctrl) cells. Left panel: One of three representative Western blots. Right panel: Quantification of the p53 signal in Western blots, normalized to the loading control GAPDH. The data represent the mean +/-SEM, *n*=3. ^*^p<0.05 (paired *t* test).

The tumor suppressor p53 has been implicated in regulation of autophagy, in particular related to cellular stress [17, 20]. It is generally believed that nuclear levels of p53 promote autophagy, whereas cytosolic levels prevent the autophagy process [20, 33]. Having previously established that cAMP-mediated inhibition of DNA damage-induced apoptosis of BCP-ALL cells involves down-regulation of p53 [24, 25], we here confirmed the ability of forskolin to reduce the level of IR-induced p53 commencing as early as 4 hours after IR (Supplementary Figure 4A). In order to assess whether the subcellular localization of p53 was affected by any of the treatments, we performed confocal microscopy of REH cells stained with an antibody directed against p53. As shown in Figure 7A, we found that IR enhanced both the nuclear and cytosolic levels of p53, whereas co-treatment with forskolin selectively reduced the levels of p53 in the nuclei. Forskolin alone had no effect on the nuclear localization of p53 (Figure 7A and 7B). To confirm these findings, we performed cellular fractionation experiments followed by immunostaining of p53. The data presented in Figure 7C confirm that forskolin selectively inhibits accumulation of p53 within the nuclei of irradiated REH cells.

**Figure 8:**
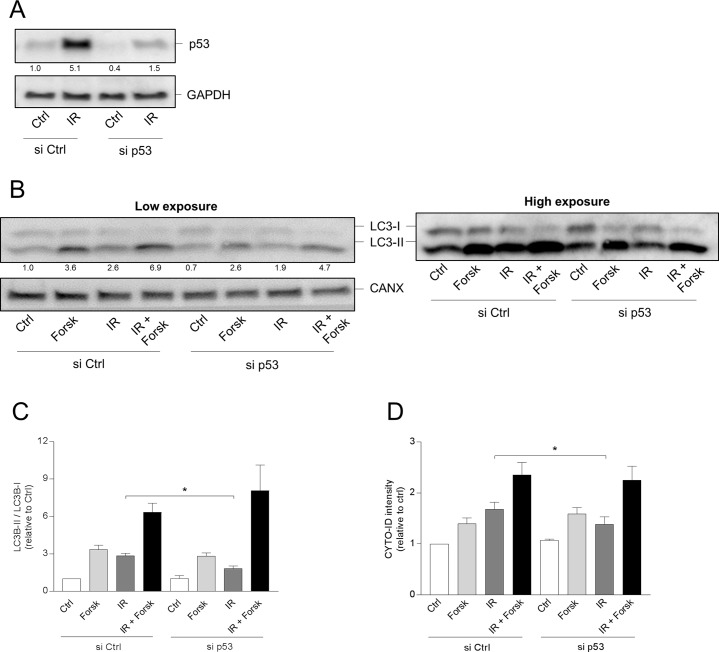
p53 is involved in IR-, but not in cAMP-induced autophagy **(A-D)** REH cells (4×10^6^ cells) were transfected with siRNA against p53 (or with scrambled siRNA as control) as described in Materials and Methods, and after 12 hours the cells were treated with or without forskolin and IR as described in Figure 1. (A) Knock-down of p53 by siRNA demonstrated by Western blot analyses of untreated (Ctrl) and irradiated (IR) cells. Signal intensities of p53 relative to GAPDH, normalized to Ctrl, are indicated as numbers below the p53 images. (B-D) BafA1 (2 nM) was added for the last 4 hours of the 24 hours incubations. (B) 24 hours after IR, the cells were harvested for Western blot analyses of LC3-II/I ratios, and one representative of 4 independent experiments is shown. The numbers indicated below the LC3 images represent the LC3-II/LC3-I signal ratios relative to the CANX signals, normalized to the ratio in untreated (Ctrl) cells. (C) Quantifications of the Western blots in panel B, presented as the ratios of LC3-II/LC3-I signal intensities relative to the CANX signals, normalized to the ratio in untreated (Ctrl) cells. The data represent the mean +/- SEM, *n*=4. ^*^p<0.05 (paired *t* test). (D) The same cells as in panel A were subjected to CYTO-ID staining, and the fluorescence intensity was analysed by flow cytometry 24 hours after IR. The data represent the mean CYTO-ID fluorescence intensity +/- SEM, *n*=4. ^*^p<0.05 (paired *t* test).

To unravel the link between p53 and autophagy in our experimental settings, p53 was targeted by siRNA. The knock-down of p53 by siRNA is demonstrated by Western blot analysis in Figure 8A. Supporting a stimulatory role in autophagy, siRNA against p53 reduced the IR-induced autophagy as revealed by the reversion of the LC3 II/I ratio (Figure 8B and 8C) and by CYTO-ID staining (Figure 8D). Furthermore, siRNA also reduced the IR-mediated cell death (Supplementary Figure 4, panel B). However, the cAMP-mediated enhancement of IR-induced autophagy could not be explained by changed localization of p53. First of all, the nuclear and not the cytosolic levels of p53 were reduced by the co-treatment with forskolin (see Figure 7). Secondly, siRNA against p53 had no effect on the ability of forskolin to enhance the IR-induced autophagy (Figure 8B-8D).

## DISCUSSION

We have previously established an *in vitro* model of BCP-ALL, successfully used for studying the interplay between p53 levels and DNA damage-induced cell death related to development and treatment of this disease [24–27]. In the present study, we extend this model to unravel a novel p53-independent interplay between autophagy and cell death with implications for treatment of BCP-ALL.

Suppression of normal p53 functions is regarded as a prerequisite for the development of most cancers [34]. Thus, mutations in the *TP53* gene itself or in p53-regulating genes render the malignant cells resistant to control mechanisms that are part of the normal DNA damage response [35]. As most childhood BCP-ALLs retain wild type TP53 at diagnosis [36], one may assume that these leukemic cells depend on alternative strategies to mitigate the function of wild type p53. We have previously suggested that stimulation of the cAMP signaling pathway may represent such a mechanism, since elevated levels of cAMP in BCP-ALL blasts suppress DNA damage-induced p53 accumulation and apoptosis [26]. Here we demonstrate that cAMP signaling also enhances DNA damage-induced autophagy in BCP-ALL blasts, enabling us to reveal the interplay between autophagy and apoptosis in these cells, and to dissect the role of p53 in these processes.

**Figure 9:**
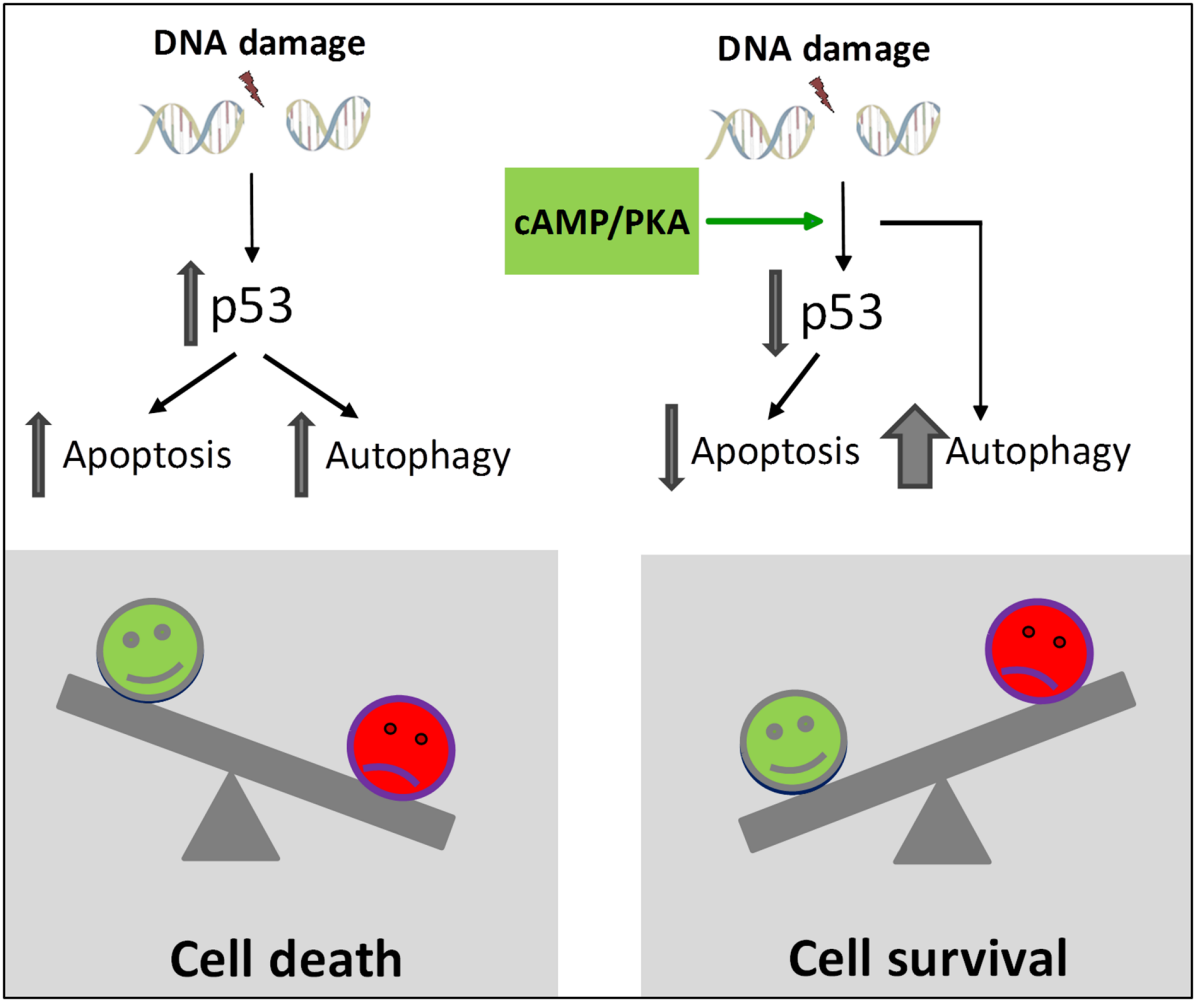
Proposed model for cAMP-mediated survival of DNA damage-induced BCP-ALL cells According to the model, cAMP-mediated survival of BCP-ALL cells exposed to DNA damage involves reduced p53-mediated apoptosis as well as p53-independent enhancement of autophagy. We suggest that autophagy needs to exceed a certain threshold in order to let the cells survive exposure to DNA damaging agents.

In most cell types, DNA damage will induce both autophagy and apoptosis [14]. However, there is no consensus as to whether the induced autophagy is required for the apoptosis or actually has a protective role [10, 13, 14]. We found that treatment of BCP-ALL cells with IR or doxorubicin promoted autophagy and killed the cells. These effects were notable in the cell line REH, as well as in primary leukemic blasts isolated from children with BCP-ALL. Blocking autophagy by BafA1 or the ULK-inhibitor MRT68921 had little or no effect on the IR-induced killing of the BCP-ALL cells. Thus, although increased autophagy has been linked to unfavorable clinical outcome of DNA damaging cancer treatments of patients with lymphoid malignancies [17], our results suggest that the level of autophagy induced by DNA damaging agents *in vitro* is too low to protect the BCP-ALL cells from the lethal DNA lesions. It was therefore interesting to find that blocking autophagy by BafA1 or the ULK1 inhibitor MRT68921 diminished the protective effect of cAMP signaling on DNA damage-induced cell death, and consequently enhanced the killing of the cells. Our results support the hypothesis that autophagy may precede apoptosis in an attempt to make the cells sustain cellular stress [14, 37]. According to our model (Figure 9), autophagy needs to exceed a certain threshold for the BCP-ALL cells to survive DNA damaging exposure. We believe that cAMP signaling enhances the level of autophagy above this threshold. A similar protective effect of autophagy was noted in bortezomib-treated BCP-ALLs, demonstrated by the enhanced cytotoxicity of bortezomib in the presence of autophagy inhibitors [38].

How then is autophagy induced by cAMP signaling in BCP-ALL cells? Our present results suggest that cAMP-mediated activation of PKA in BCP-ALL cells is sufficient to promote autophagic flux both alone and in the presence of irradiation. Furthermore, the involvement of ULK1 in the process was proven by the ability of either the ULK inhibitor MRT68921 or siRNA against ULK1 to prevent the treatment-enhanced autophagy. However, we did not observe changes in activation of common autophagy inducers such as AMPK, ULK1, beclin1, or other autophagy-related proteins that could explain our results. We are currently pursuing these investigations, but here we turned to the ubiquitin-binding scaffold protein SQSTM1/p62. SQSTM1/p62 is a common marker of autophagic flux, and the protein is frequently degraded as part of the autophagy process [39]. In line with this notion, we observed that whereas irradiation reduced the levels of SQSTM1/p62 (Supplementary Figure 5A), BafA1 did not prevent the IR-mediated downregulation of SQSTM1/p62 (S5A, right panel). Furthermore, forskolin opposed the IR-induced decline in SQSTM1/p62 by enhancing the transcription of the *SQSTM1* gene (S5B). Taken together, we do not believe that regulation of SQSTM1/p62 can explain the IR and/or cAMP-mediated induction of autophagy seen in the present study.

One of the suggested mediators of DNA damage-induced autophagy is p53 [20, 21]. The mechanisms whereby p53 promotes autophagy is not fully understood, but it appears that p53 induces the transcription of autophagy-related genes such as AMPK β1/β2, DRAM and DAPK-1 [40, 41]. Accordingly, we observed reduced autophagy when the level of p53 in irradiated cells was downregulated by siRNA. However, a simplistic view on the association between p53 levels and autophagy was challenged by the notion that even more autophagy was detected concomitant with reduced levels of p53 when the BCP-ALL cells were co-exposed to irradiation and activators of the cAMP signaling pathway. Although reports have suggested that lowering the levels of p53 may facilitate autophagy [42, 43], the consensus has been that the subcellular localization of p53 dictates its role in autophagy. Thus, induction of nuclear p53 is assumed to promote autophagy, whereas p53 in the cytosol protects the cells from autophagy [20, 21, 33]. We found that IR alone resulted in enhanced levels of p53 both in the nuclei and in the cytoplasm, and we postulated that cAMP signaling might selectively lower the cytoplasmic level of p53. This turned out not to be the case; in fact, cAMP signaling completely diminished the p53 levels in the nuclei, while preserving the levels in the cytosol. Furthermore, siRNA against p53 did not affect the cAMP-mediated enhancement of irradiation-induced autophagy. Taken together, our results suggest that cAMP signaling enhances DNA damage-induced autophagy in a p53-independent manner.

We have previously shown that bone marrow-derived stromal cells provide BCP-ALLs with cAMP-stimulating PGE2, and that cAMP signaling in turn reduces DNA damage-induced p53 and apoptosis in the leukemic blasts [27]. This led us to propose that cyclooxygenase (cox)-inhibitors or other inhibitors of the cAMP signaling pathway might improve DNA damage-based therapy of BCP-ALL by sustaining p53-mediated apoptosis [27]. Based on our current results, we propose that therapies targeting the cAMP signaling pathway also might reduce the level of autophagy in the leukemic cells, and in this manner increase the killing of the cells. Furthermore, our results imply that therapies directed against the autophagy machinery itself might improve DNA damage-based treatment of BCP-ALLs. Having shown that cAMP signaling enhances autophagy of BCP-ALL cells in a p53-independent manner, we propose that such therapies not only might improve the treatment of BCP-ALLs with wild type *TP53* genes, but might also improve the therapy of BCP-ALL cases harboring *TP53* mutations. Mutations in *TP53* are common in adult BCP-ALLs [44], and although rare at diagnosis of pediatric BCP-ALL, the frequency increases with relapse [45, 46]. BCP-ALL patients with *TP53* mutations generally respond poorly to current therapies [45, 47]. We therefore suggest that targeting the autophagy machinery, with for instance the newly developed small molecule inhibitors of ULK1 [48, 49], could be particularly valuable for treatment of this group of BCP-ALL patients.

## MATERIALS AND METHODS

### Reagents and antibodies

Forskolin, doxorubicin, propidium iodide (PI), paraformaldehyde (PFA), and 3-methyladenine (3-MA) were purchased from Sigma-Aldrich. 8-CPT-cAMP and RP-8-Br-cAMPS were from BioLog, and bafilomycin A1 (BafA1) was from AH Diagnostics. The ULK1 inhibitor MRT68921 was obtained from Selleckchem. Antibody against p53 (DO-1, # SC-126) was obtained from Santa Cruz Biotechnology. Anti-LC3B (# 2775) used for Western blot analyses was purchased from Cell Signaling Technology, whereas anti-LC3B (# PM036) used for immunofluorescence analyses was obtained from MBL International. Antibodies against glyceralaldehyde 3-phosphate dehydrogenase (GAPDH) was purchased from Sigma Aldrich. As loading control in Western blot analyses we used an antibody directed against Calnexin (# 2433) from Cell Signaling Technology.

### Cell culturing and primary cell isolation

The B-cell precursor acute lymphoblastic leukemia cell lines REH [50] and NALM-6 [51] were kept at a density between 0.2 × 10^6^ and 1.0 × 10^6^ cells /ml, and the cells were cultured as described [52]. Primary leukemic blasts were isolated from bone marrow aspirates of three children with BCP-ALL as previously described [24]. The proportion of BCP-ALL blasts was 73%, 40% and 90% for cells derived from patient #1, #2 and #3, respectively (Table 1), as assessed by co-staining of cells with antibodies against CD19 and CD10 [24]. The collection of bone marrow aspirates was performed after informed consent by parents, in accordance with the Declaration of Helsinki. The collection of material was approved by the Regional Ethics Committee of Norway region Sør-Øst C (REK 2014/883).

**Table 1:** Patient characteristics

	Patient #1	Patient #2	Patient #3
Age, y	8	9	9
Sex	M	M	F
Bone marrow infiltration at diagnosis (% CD19+ / CD10+)	73%	40%	90%
Cytogenetics	48,XY,+X,+21 [2]/46,XY [23]	Hyperdiploidy	Normal karyotype

### Irradiation

Cells were irradiated using an Xstrahl RS320 X-ray irradiator at a rate of 3.9 Gy/min. REH cells were irradiated at 10Gy, whereas primary BCP-ALL blasts from patient were irradiated at 5Gy.

### Analyses of autophagy

#### LC3-II/I ratio

To determine autophagic flux, BafA1 was added to the samples to block lysosomal degradation, resulting in the accumulation of autophagosomes [8]. The levels of cytosolic LC3B (LC3I) and LC3B bound to autophagosomal membranes (LC3-II) were normalized to the loading control as estimated by Western blot analysis (see below), and the LC3-II/I ratios were calculated.

#### LC3B puncta analyses

LC3B puncta characteristic of autophagosomes [9] were visualized by immunocytochemistry and confocal microscopy as described below, after staining the cells with antibodies against LC3B or with Hoechst for visualizing the nuclei.

#### CYTO-ID staining

Autophagy was also measured by the CYTO-ID® Autophagy detection kit (Enzo Life Sciences, Farmingdale, NY, USA), according to manufacturer's protocols. Stained cells were analysed both by immunocytochemistry and by flow cytometry (see below).

*Degradation of long-lived proteins* was performed essentially as previously described [53]. In brief, the degradation of short-lived proteins was allowed by seeding REH cells in RPMI medium (Lonza) containing 10% FBS and 0.25 μM Ci/ml L-^[14C]^ valine (Perkin Elmer) for 24 hours, before the cells were washed and chased for another 24 hours in RPMI containing 10% FBS and 10 mM valine (Sigma). The cells were then treated with or without irradiation and forskolin for 22 hours, before washing and further incubating the cells for 4 hours in starvation medium (EBSS) in the presence or absence of the autophagy inhibitor 3-MA (5mM). Finally, autophagic flux was determined by subtracting the degradation of long-lived proteins in cells cultured in the presence of 3-MA from that of cells cultured in the absence of 3-MA, as described [53].

### Western blot analysis

Cells were harvested and lysed in radioimmunoprecipitation (RIPA) buffer as previously described [54]. Equal amounts of proteins were separated by SDS-PAGE gel electrophoresis (Bio-Rad). Proteins were transferred to an Immobilion-P transfer membrane (Merck Millipore), and detected using standard immunoblotting techniques. Proteins were visualized using SuperSignal™ West Dura Extended Duration Substrate kit (Thermo Fisher Scientific) according to the manufacturer's instructions. Images were captured using a Syngene ChemiGenious camera and presented by the GeneSnap software tool (Syngene, Cambridge, England). Intensity of protein bands was quantified by using the GeneTool software (Syngene).

### Immunofluorescence staining and confocal microscopy

#### Analyses of LC3 puncta and subcellular localization of p53

REH cells (3,5x 10^4^ cells per slide) were adhered to poly-L-lysine coated microscopic slides by cytocentrifugation at 370 x g for 4 min. The cells on slides were fixed in 4% PFA for 15 min at room temperature. For analyses of LC3 puncta, the cells were permeabilized with saponin (0.05%) followed by blocking in 2% FBS/PBS for 30 min. The cells were then incubated with antibody against LC3B (PM036, MBL) over-night at 4°C, followed by incubation with Alexa488-conjugated donkey anti-rabbit IgG (A-21206, Thermo Fisher Scientific) for 1 hour at room temperature. Subcellular localization of p53 was determined by incubating the slides over-night with anti-p53 (FL-393Santa Cruz), followed by incubation for 1 hour at room temperature with Cy-3 AffiniPure goat anti-rabbit IgG from Jackson Immunoresearch. For visualization of nuclei, the cells were stained with Hoechst 33258 (1 μg/ml in PBS) from Sigma Aldrich. Fiji was used for quantifying the p53 signals.

#### Co-staining between LC3 and CYTO-ID

Living cells were stained with CYTO-ID (Enzo Life Sciences, Farmingdale, NY, USA) according to manufacturer's recommendations. Stained cells were resuspended in RPMI starvation media for 15 min at 4°C, allowing cells to attach to the poly-L-lysine coated microscopic slides. The cells were then fixed in 4% PFA, permeabilized with saponin, and stained with antibody against LC3 as described above. Images were acquired using a Confocal Laser Scanning microscope (LSM 710, Axio Observer, Carl Zeiss Inc.), equipped with 63 × 1.4 NA oil immersion objective, and the images were processed using the ZEN software.

### Flow cytometry

All flow cytometry analyses were performed on a FACS Calibur instrument (BD Biosciences). For cell death analysis, cells were incubated with propidium iodide (PI) (20 μg/ml) for 10 min at 4°C. Quantification of autophagy was performed by using the CYTO-ID® Autophagy detection kit, according to the manufacturer's protocol. Data were analyzed using the CellQuest software (BD Biosciences).

### Transfection of small-interfering RNA oligonucleotides

REH cells (4×10^6^) were transfected with small-interfering RNA (siRNA) by using a nucleofector device (Amaxa Biosciences) and the Nucleofector^®^ Kit R (Lonza) according to the manufacturer's instructions and using the program G-009. For knock-down of ULK1 and p53 we used 1.6 μM of ULK1 siRNA (L-005049-00-0010) or p53 siRNA (L-003329-00-0010). A non-targeting siRNA (D-001810-01-05) was used as control. All siRNAs were obtained from Dharmacon. After transfection, the cells were incubated for 12 hours before further treatments were initiated.

### Fractionation of cytoplasms and nuclei

REH cells (20×10^6^) were harvested 4 hours after IR and resuspended in Hypotonic buffer (10mM Tris-HCl pH 7.6, 10mM NaCl, 3mM MgCl_2_), NP-40 was added to a final concentration of 0.05%. The nuclei were collected for Western blot analyses by centrifugation at 200 x g for 5 min, lysed in 2% SDS, and sonicated. The supernatants (cytoplasmic fractions) were used directly for Western blot analysis.

### Analyses of transcription of *MAP1LC3B* and *SQSTM1* by real-time quantitative PCR

Total RNA was isolated from REH cells (1.5 × 10^6^) 24 hours after treatment with forskolin and irradiation, using the RNeasy plus mini kit (QIAGEN) according to manufacturer's instructions. cDNA (800 ng) was synthesized by reverse transcription (iScript; Bio-Rad technologies), and qPCR was performed using SsoFast^™^ EvaGreen^®^ Supermix (Bio-Rad technologies). The level of *MAP1LC3B* and *SQSTM1* transcripts were normalized to the housekeeping genes; TATA binding protein (*TBP*) and β2-microglobulin (*B2M)* by using the 2^-ΔCt^ method. Primers from Qiagen were: *TBP*: QT00000721, *B2M*: QT00088935, *MAP1LC3B:* QT01750322, and *SQSTM1:* QT00095676.

### Statistics

GraphPad Prism 7 was used to perform statistical analyses. The paired *t* test was used to investigate for statistical significant differences. Unless otherwise stated, graphs are presented as mean values from at least three independent experiments, with error bars indicating the standard error of the mean (SEM).

## SUPPLEMENTARY MATERIALS FIGURES


