# Supplementary material for: cAMP-mediated autophagy inhibits DNA damage-induced death of leukemia cells independent of p53
Source: Oncotarget. 2018 Jul 13;9(54):30434–49. doi: 10.18632/oncotarget.25758 (PMC6084393; doi:10.18632/oncotarget.25758)
Supplement: Supplementary file 1 [file oncotarget-09-30434-s001.pdf]

cAMP-mediated autophagy inhibits DNA damage-induced death of leukemia cells independent of p53

SUPPLEMENTARY MATERIALS

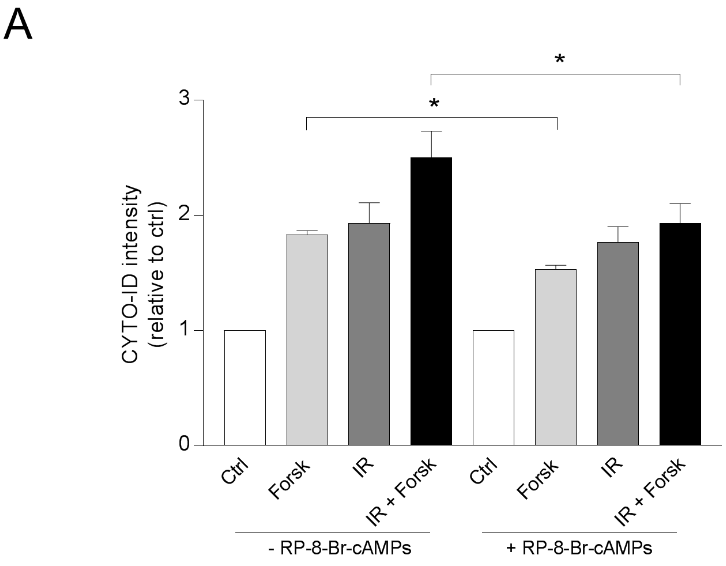

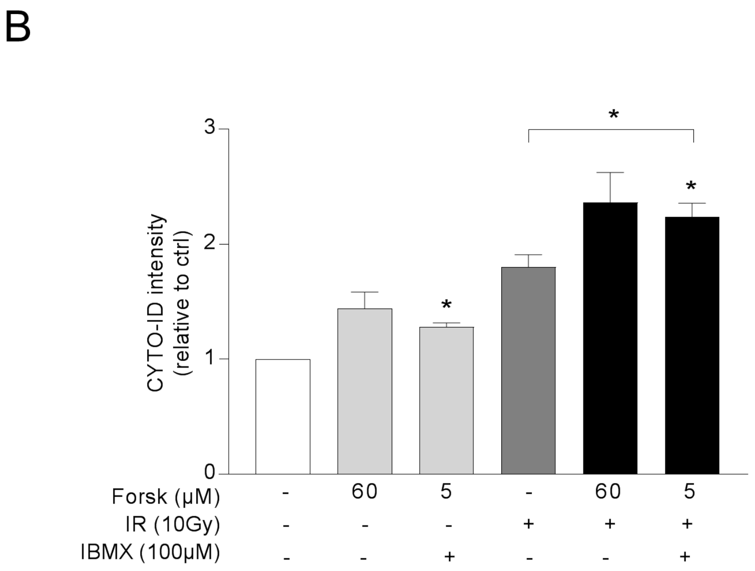

Supplementary Figure 1: Forskolin-mediated activation of DNA damage-induced autophagy is mediated via PKA. REH cells ($0.5x10^6$) were treated with forskolin (Forsk, 60μM) for 45 minutes prior to irradiation (IR, 10Gy). CYTO-ID staining was performed 24 hours after IR, and analyzed by flow cytometry. **(A)** REH cells were treated with RP-8-Br-cAMPs (1mM) for 30 minutes prior to treatment with forskolin. The data represent the mean CYTO-ID fluorescence intensity +/-SEM, *n*=3. $^*p<0.05$ (paired *t* test). **(B)** REH cells were treated with IBMX (100μM) for 30 minutes prior to treatment with forskolin (5μM). The data represent the mean CYTO-ID fluorescence intensity +/-SEM, *n*=5. $^*p<0.05$ (paired *t* test).

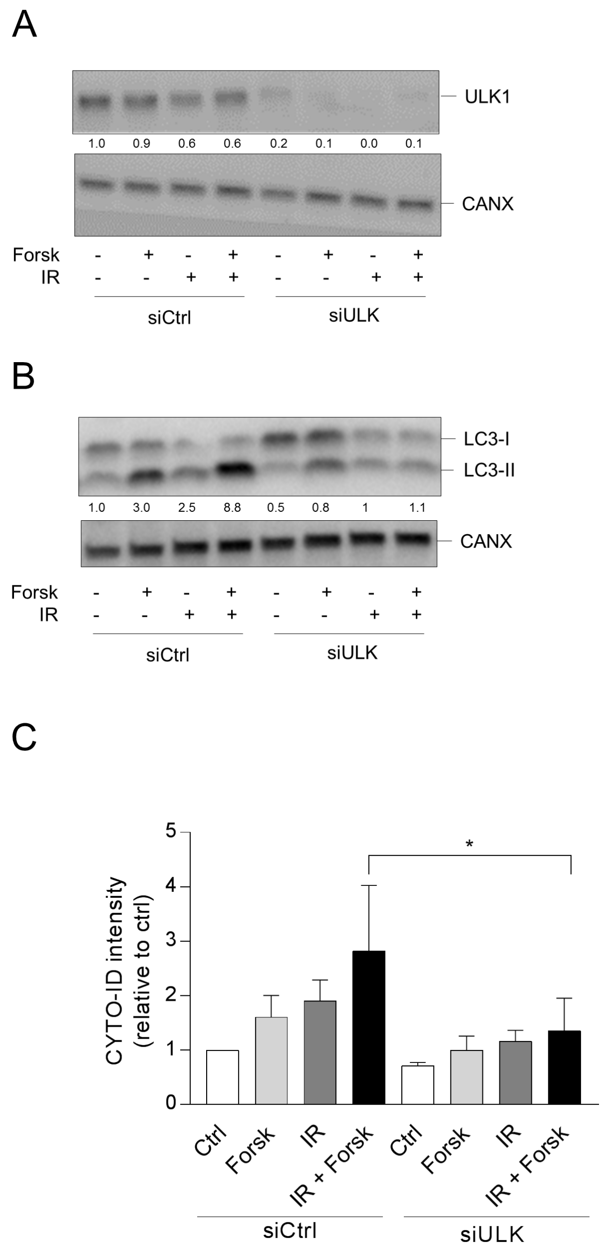

Supplementary Figure 2: The effect of ULK1 siRNA on autophagy. REH cells ($4x10^6$) were transfected with siRNA against ULK1 or with control siRNA as described in Materials and Methods. After 12 hours, the cells were treated with or without forskolin (Forsk, 60μM) for 45 minutes before irradiation (IR, 10Gy). The cells were harvested after 24 hours and subjected to Western blot analyses (Panel A and B) or subjected to CYTO-ID staining (panel C). **(A)** Knock-down of ULK1 demonstrated by Western blot analysis. The numbers indicated below the ULK1 images represent the ULK1 signal relative to the CANX signal, normalized to the signal in untreated (Ctrl) cells. One representative Western blot of three experiments is shown. **(B)** The effect of ULK1 siRNA on LC3-II/I ratios. The numbers indicated below the LC3 images represent the LC3-II/LC3-I signal ratios relative to the CANX signals, normalized to the ratio in untreated (Ctrl) cells. One representative Western blot of four experiments is shown. **(C)** CYTO-ID staining intensity analysed by flow cytometry. The data represent the mean CYTO-ID fluorescence intensity +/- SEM, *n*=4. $^{*}p<0.05$ (paired *t* test).

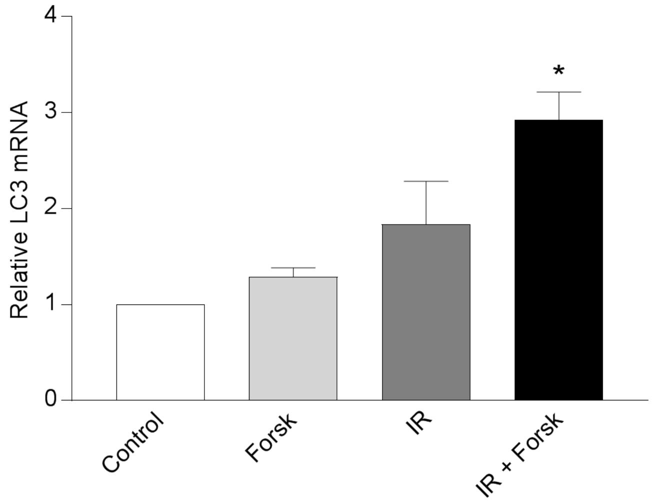

Supplementary Figure 3: RTq-PCR for LC3. REH cells were treated with forskolin (Forsk, 60µM) prior to irradiation (IR, 10Gy), and total RNA was isolated from the cells 24 hours after IR. RTqPCR was performed as described in Materials and Methods, using primers for the *MAP1LC3B* gene. The data were normalized to the housekeeping genes *TBP* and *B2M.* The results are presented as the expression of *MAP1LC3B* mRNA relative to the levels in untreated (Ctrl) cells. The data represent the mean +/- SEM, n=3. $^{*}p<0.05$ (paired t test).

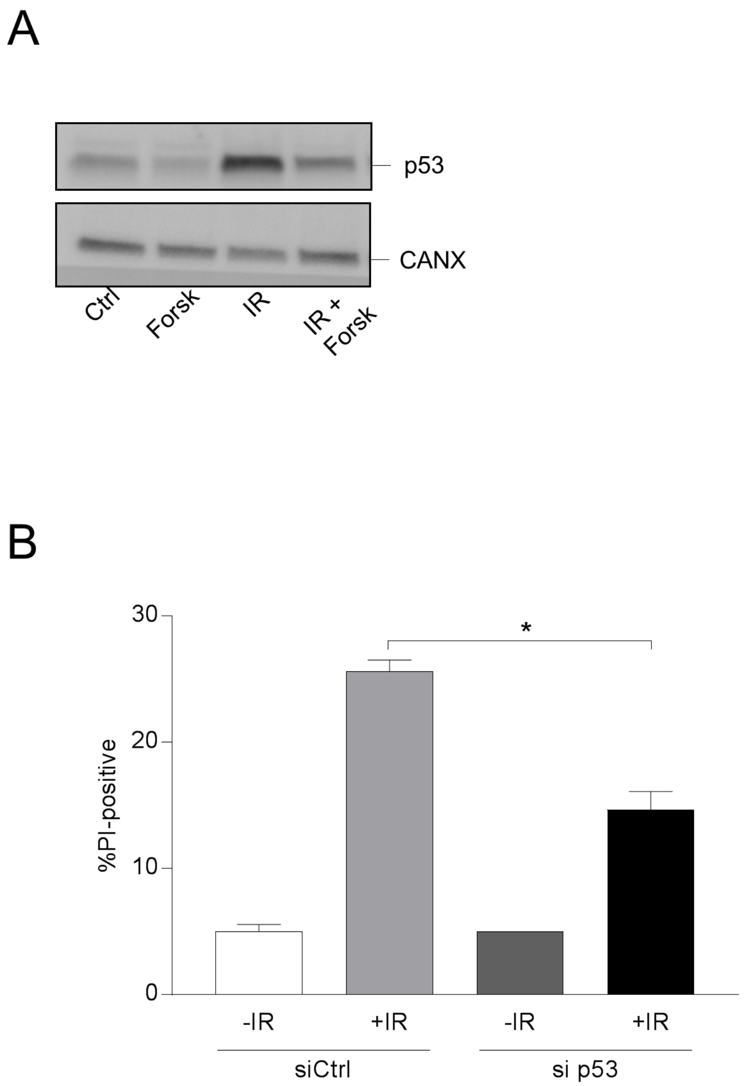

Supplementary Figure 4: Knock-down of p53 on cell death in BCP-ALLs. (A) REH cells ($2x10^6$) were treated with or without forskolin (Forsk, 60 µM) for 45 minutes prior to irradiation (IR, 10Gy). The cells were harvested 4 hours after IR, and subjected to immunoblot analysis with antibodies against p53 or calnexin (CANX). One representative Western blot of at least 10 experiments is shown. **(B)** REH cells were transfected with siRNA against p53 or with control siRNA as described in Materials and Methods. 12 hours after transfection, the cells were treated with or without IR (10Gy) as indicated, followed by PI-staining 24 hours after IR. The data represent the percentage of PI-positive cells and are presented as the mean +/- SEM, *n*=3. $^*p<0.05$ (paired *t* test).

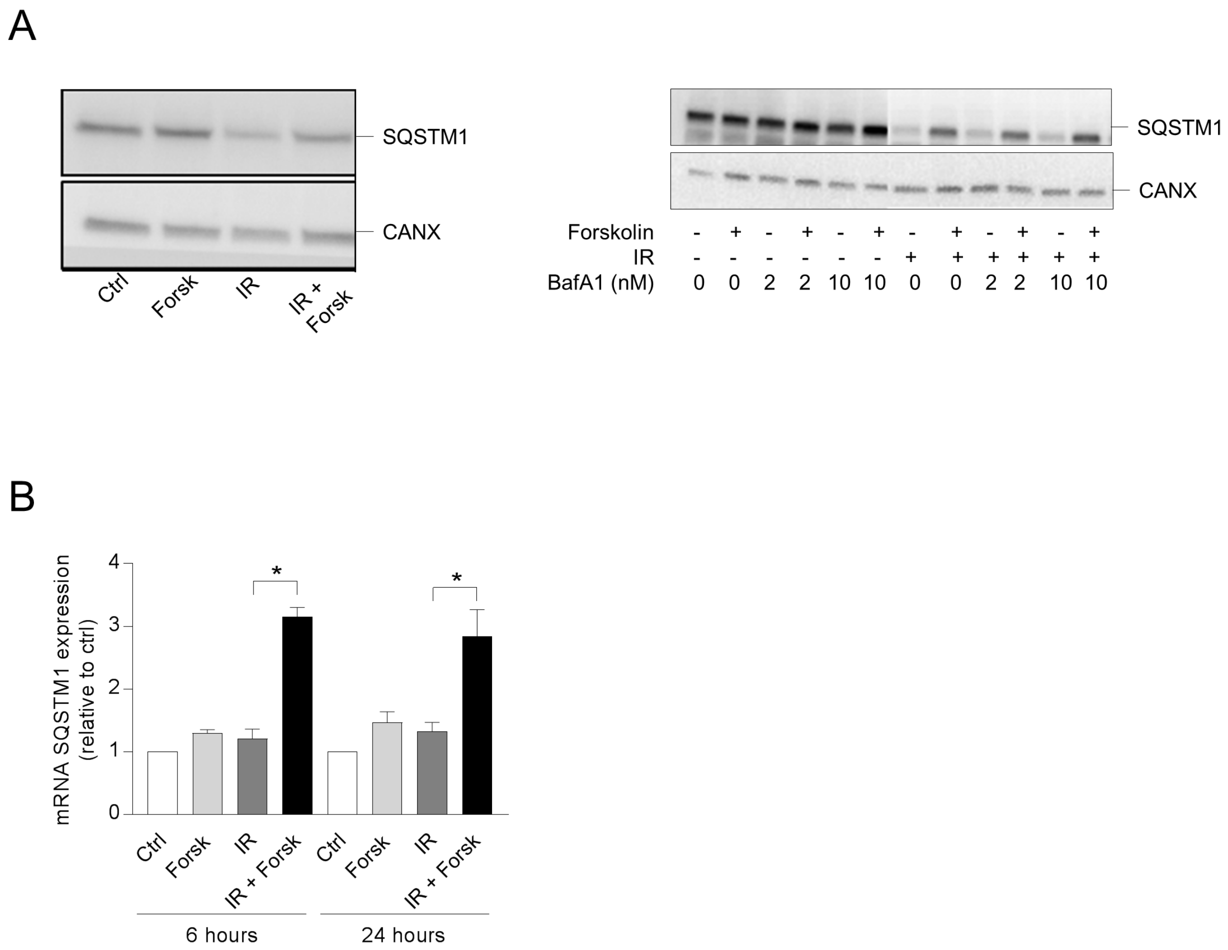

Supplementary Figure 5: Regulated expression of SQSTM1/p62. REH cells were treated with or without forskolin (Forsk, 60µM) for 45 minutes prior to irradiation (IR, 10Gy). **(A)** Left panel: The cells were subjected to immunoblot analyses of the LC3II/I ratios 24 hours after IR. Right panel: BafA1 at the indicated concentrations was added to the cell cultures 4 hours prior to harvesting the cells. Each panel shows one representative Western blot of three experiments. **(B)** RNA was isolated at the indicated time points post IR, and RT-qPCR was performed as described in Materials and Methods, using primers for the *SQSTM1* gene. The data are normalized to the housekeeping genes *TBP* and *B2M* and represent the mean +/-SEM, *n*=3. $^{*}p<0.05$.
